# Effects of a single transient transfection of Ten-eleven translocation 1 catalytic domain on hepatocellular carcinoma

**DOI:** 10.1371/journal.pone.0207139

**Published:** 2018-12-14

**Authors:** Yuying Liu, Hui Zhu, Zhenxue Zhang, Changchun Tu, Dongyuan Yao, Bin Wen, Ru Jiang, Xing Li, Pengfei Yi, Jiejie Zhan, Jiaping Hu, Jianwu Ding, Liping Jiang, Fanglin Zhang

**Affiliations:** 1 College of Pharmacy, Nanchang University, Nanchang, Jiangxi, P.R. China; 2 The First Affiliated Hospital, Nanchang University, Nanchang, Jiangxi, P.R. China; 3 Jiangxi Maternal and Child Health Hospital, Nanchang, Jiangxi, P.R. China; 4 Gannan Medical University, Ganzhou, Jiangxi, P.R. China; 5 Jiangxi Provincial Children's Hospital, Nanchang, Jiangxi, P.R. China; 6 The Second Affiliated Hospital, Nanchang University, Nanchang, Jiangxi, P.R. China; Chinese University of Hong Kong, HONG KONG

## Abstract

Tumor suppressor genes (TSGs), including Ten-eleven translocation 1 (TET1), are hypermethylated in hepatocellular carcinoma (HCC). TET1 catalytic domain (TET1-CD) induces genome-wide DNA demethylation to activate TSGs, but so far, anticancer effects of TET1-CD are unclear. Here we showed that after HCC cells were transiently transfected with TET1-CD, the methylation levels of TSGs, namely APC, p16, RASSF1A, SOCS1 and TET1, were distinctly reduced, and their mRNA levels were significantly increased and HCC cells proliferation, migration and invasion were suppressed, but the methylation and mRNA levels of oncogenes, namely C-myc, Bmi1, EMS1, Kpna2 and c-fos, were not significantly change. Strikingly, HCC subcutaneous xenografts in nude mice remained to be significantly repressed even 54 days after transient transfection of TET1-CD. So, transient transfection of TET1-CD may be a great advance in HCC treatment due to its activation of multiple TSGs and persistent anticancer effects.

## Introduction

Hepatocellular carcinoma (HCC) is one of the most malignant tumors with an increasing incidence and mortality rate [1]. Unfortunately, ideal drugs for HCC treatment are lacking due to multi-drug resistance and liver toxicity of systemic chemotherapeutic drugs [2]. So, it is urgently required to find a more effective way for HCC treatment.

HCC initiation and progression are triggered by multiple factors, which include hepatitis B and C virus infection, chronic alcohol consumption, non-alcoholic fatty liver disease, and cirrhosis. The fundamental mechanism of carcinogenesis has long been recognized as activation of oncogenes and/or deactivation of tumor suppressor genes (TSGs) [3], which are closely related to demethylation and hypermethylation, respectively [4–8]. Indeed, hypermethylation of TSG promoter CpG islands is a ubiquitous phenomenon in human tumors [9, 10]. Therefore, it is possible that demethylation can be used to activate the hypermethylated TSGs to repress human tumors.

A methyl group is transferred from a methyl donor S-adenosylmethionine by DNA methyltransferase (DNMT) to methylate the C-5 position of cytosine in promoter CpG islands to form 5-methylcytosine (5-mc), which silences TSGs. The process of demythylation starts once 5-mc is consecutively converted into 5-hydroxymethylcytosine (5-hmc), 5-formylcytosine (5-fc) and 5-carboxylcytosine (5-cac) through the dioxygenase action of ten eleven translocation (TET) protein [11–13]. When 5-fc and 5-cac are identified and removed by thymine DNA glycosylase (TDG) [14], demethylation is accomplished.

It has been confirmed that the promoter CpG islands of TSGs, such as genes of adenomatous polyposis coli (APC), p16, isoform A of RAS association domain family 1 (RASSF1A) and suppressor of cytokine signaling1 (SOCS1), are hypermethylated [15–21]. The existence of hypermethylated promotors might be related to TET1 since TET1 can make them be demethylated. However, TET1 expression was down-regulated in HCC [22–24], which is related to hypermethylation of the TET1 promoter CpG islands [25]. Given that the TET1 catalytic domain (TET1-CD) induces genome-wide DNA demethylation while full length TET1 does not [26], it is likely that through demethylation, TET1-CD activates endogenous TET1 and then endogenous TET1 activates TSGs to exert anticancer effects. Therefore, the study was aimed to test whether TET1-CD had anti-HCC effects in vitro and in vivo.

## Materials and methods

### Cell lines and cell culture

Human normal liver cell line (LO2) and hepatocellular carcinoma cell line (SMMC 7721 cells) were obtained from the CRC cell bank (Shanghai, China) and have been identified by STR analysis on April 20, 2017, incubated in the RPMI-1640 medium (BI, Israel) under 5% CO2 atmosphere supplemented with 10% fetal bovine serum (FBS) (Sigma) at 37°C.

### Plasmid construction

In order to get TET1 catalytic domain (TET1-CD, codons 1418 to 2136) cDNA, total RNA was extracted from the LO2 cells using Trizol reagent (Invitrogen), and reversed into cDNA using PrimeSTAR HS DNA Polymerase (TARAKA), then subcloned into pflag-CMV4 vector digested with Not I and Bgl II restriction enzymes. The TET1 catalytic domain mutant (TET1-mCD) (H1672Y, D1674A) was generated by site-directed mutagenesis. The sequences of plasmids were validated by Sanger DNA sequencing. The primers used were list S1 Table.

### Cell transfection

The SMMC 7721 cells were cultured in 6 mm plates for 24h and were then transiently transfected with pflag-CMV4 vector (control group), TET1-CD (TET1 group) or TET1-mCD (TET1-mCD group), and each group were repeated 3 times. All transfections were formed using Lipofectamine 3000 reagent (Invitrogen) according to the manufacturer’s instructions.

### RNA extraction and quantitative real-time PCR

Total RNA was extracted from the cells using Trizol reagent (Invitrogen) according to the manufacturer’s protocol. The concentration and purity of RNA were determined by the absorbance at 260 nm and the ratio of 260/280, respectively, on a nucleic acid quantitative analyzer(Thermo Fisher Scientific). Total RNA (1 ug) was reversely transcribed into cDNA using GoScript Reverse Transcription System(Promega), and qPCR was performed using GoTaq qPCR Master Mix (Promega). All primer sequences were listed in S1 Table.

### Western blot

The cells were collected by radio-immunoprecipitation assay (RIPA) buffer (Thermo Fisher Scientific) containing 1x protease inhibitor cocktail solution(Roche). The total protein concentration was measured using the BCA protein assaykit(Bio-Rad). The proteins were separated by 10% SDS-PAGE and transferred onto PVDF membrane. Then, the membrane was blocked with 5% non-fat milk for 2h at room temperature, and then incubated at 4°C overnight with anti-TET1 (GeneTex) and anti-FLAG (Stratagene) antibody (1:1000 dilution), or mouse monoclonal anti-β-actin (Sigma) antibody (1:5000 dilution) for the internal control. Finally, the membrane was washed again and detected using ECL method (Thermo Fisher Scientific) by gel imaging system (Bio-Rad). The results were evaluated by the Image J software (NIH).

### Bisulfite sequencing

Bisulfite treatment of genomic DNA was completed with EZ DNA Methylation Gold Kits (Zymo Research). The bisulfate-treated DNA was amplified by PCR with primers designed by MethPrimer of the LiLab. Then PCR products were subcloned into T-Easy vector(Promega). Eightclones randomly selected from each treatment group were sent for sequencing. Sequencing results were analyzed by BiQ analyzer. Primers used for bisulfite sequencing were listed in S1 Table.

### Cell proliferation

Cell proliferation was assessed by Cell Counting Kit-8 (CCK-8) (Quanshijin) and 5-ethynyl-2-deoxyuridine (EdU) imaging assay (KeyGEN BioTECH). For the CCK-8 assay, 2×10^3^ cells were seeded per well in the 96-well plate with 100 μL of the culture medium. Finally, the absorbance at 450 nm was measured at 1, 2, 3 day after incubation at 37°C for 1 h with 10 μL of the CCK-8 solution. For the EdU imaging assay, the SMMC 7721 cells were inoculated into the 24-well plate at 4 x 10^4^ cells per well. Then, fluorescence staining was performed by use of the EdU kit according to the manufacturer’s instruction. After staining, the images were collected at 40X using inverted fluorescence microscope.

### Wound healing assay

A total of 5 × 10^5^ cells were seeded into the 6-well plate and incubated overnight to achieve 100% confluence. A straight line was drawn with a 10 μl pipette tip and cultured in the serum-free medium, and photographed at 0 h, 24h, and 48 h with a microscope to obtained cell scratched area.

### Cell migration and invasion

Transwell assay was performed to test the ability of cell migration and invasion. To measure cell migration, 1 × 10^5^ cells were seeded on the upper chamber with 200 μL serum-free medium, and 600 μL RPMI1640 medium containing 10% FBS was added to the lower chamber. After incubating for 36h, the cells in the upper chamber were wiped off with a cotton swab. Then cells that migrated out of the upper chamber were fixed with 4% paraformaldehyde and stained with crystal violet. Finally cells were captured by Olympusinverted fluorescence microscope and counted by use of ImageJ software. To measure cell invasion, the matrigel basement membrane matrix (BD) was coated on the upper chamber and 1 x 10^5^ cells were seeded on the upper chamber with 200 μL serum-free medium. The other steps were the same as the cell migration steps described above.

### Animal experiments

The procedures and protocols of animal experiments were approved by the local Animal Ethics Committee. Female nude mice (4-week-old, approximate 17 g each) were purchased from the Shanghai SLAC Laboratory Animal Corporation (Shanghai) and kept in sterile environment. The nude mice were randomly divided into 3 groups (5 samples per group): control group, TET1-CD group, TET1-mCD group. The SMMC 7721cells were suspended in PBS to a final concentration of 2 × 10^7^/ml, a total of 2 × 10^6^ cells were subcutaneously injected into each nude mouse to establish a model of subcutaneous tumor in nude mice, and the food and water intake, defecation, urination, exercise, fur color and appearance of the mice were closely monitored and the mice were weighed every two days to assess their health status. Once the tumor volume reached approximately 50 mm^3^ (V = length × width^2^/2), it was measured every 4 days. Finally, when the mice showed symptoms, e.g. weight loss more than 20%, diarrhea and infection, the nude mice were euthanized by carbon dioxide. The tumor tissues were fixed with 4% paraformaldehyde and used for further experiments.

### Immunohistochemistry

Tumor tissues were fixed for 24h and 4-μm paraffin-embedded sections were obtained for further experiments. The Ki-67 protein expression was detected using immunohistochemistry assay according to the manufacturer’s protocol. Sections of the tumor tissues were incubated with primary Ki-67 antibody (Proteintech) (1:1000 dilution) at 4°C overnight and then incubated with biotinylated secondary antibody. Diaminobenzidine (DAB) and hematoxylin were used to visualize the Ki-67 protein and counter-stained, respectively. The staining results were observed with fluorescence microscope.

### Statistical analysis

All data were presented as mean ± SD and analyzed using GraphPad Prism software. Student’s t-test and one-way ANOVA were used wherever appropriate.

## Results

### TET1 expression was suppressed in SMMC 7721 cells

**Fig 1:**
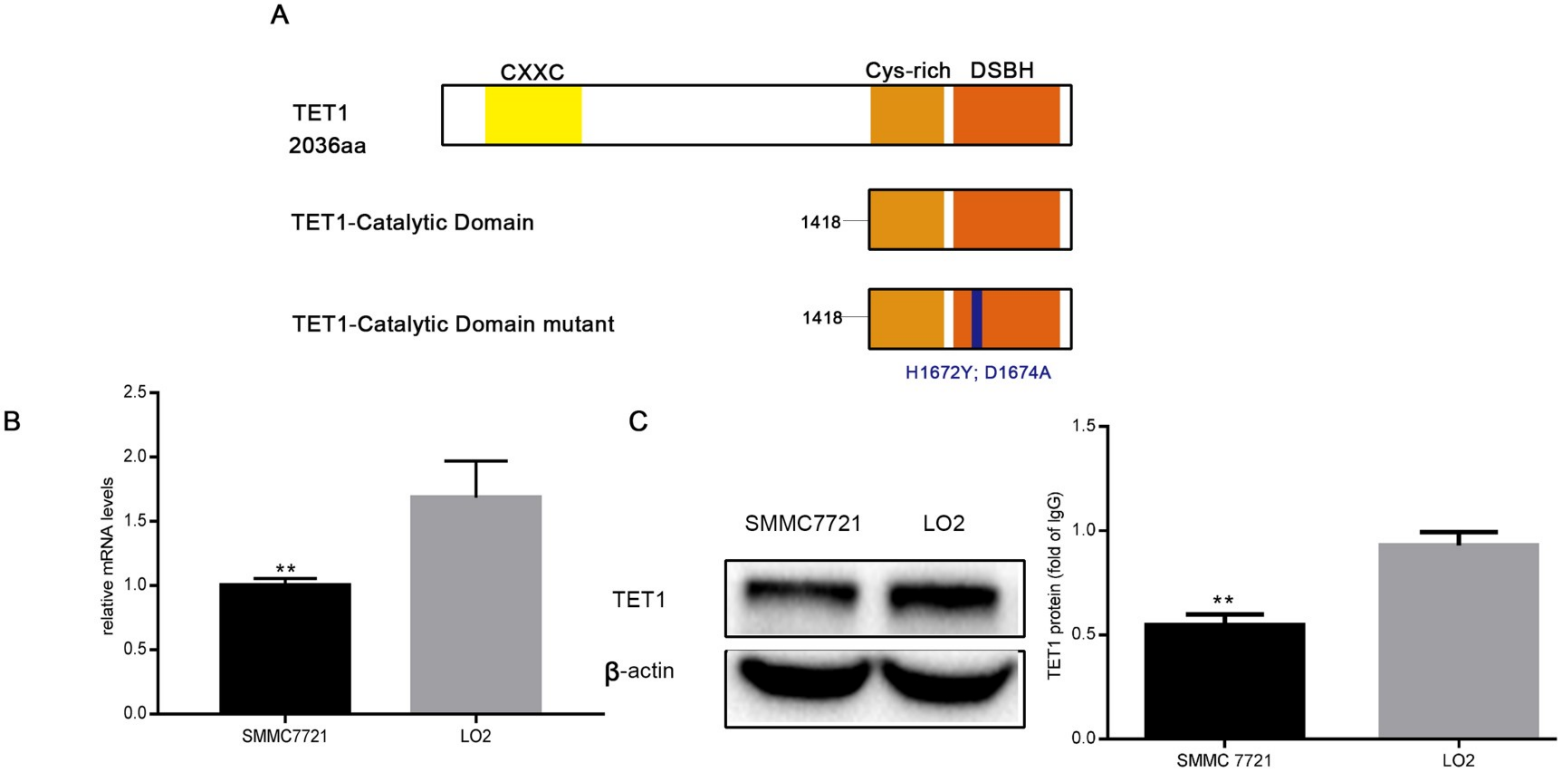
The expression of TET1 is downregulated in the SMMC 7721 cells. (A). Structure of TET1 protein. TET1 protein contains a CXXC domain and a catalytic domain (CD) including a Cys-rich part and a DSBH part. TET1-mCD (TET1-CD mutant) harbours two mutated amino acid in the DSBH part of the CD. (B). Expression of TET1 mRNA in the SMMC 7721 cells and LO2 cells was analyzed by Quantitative RT-PCR, and the results were represented as mean ± SD of three independent experiments.**p<0.01. (t-test). (C). Western blot was used to analyze the expression of TET1 protein in the SMMC 7721 cells and LO2 cells. β-actin was used as an internal control.

Expression of TET1, but not TET2 and TET3, was downregulated in HCC cells [22], likely resulting from hypermethylation of TET1 promoter CpG islands[25, 27]. In the study, the downregulation of TET1 expression in SMMC 7721 cells was again verified (Fig 1B and 1C), suggesting that application of TET1-CD transient transfection into HCC cells can be possibly to treat endogenous TET1 deficiency.

### TET1-CD demethylated TSGs and promoted its expression

**Fig 2:**
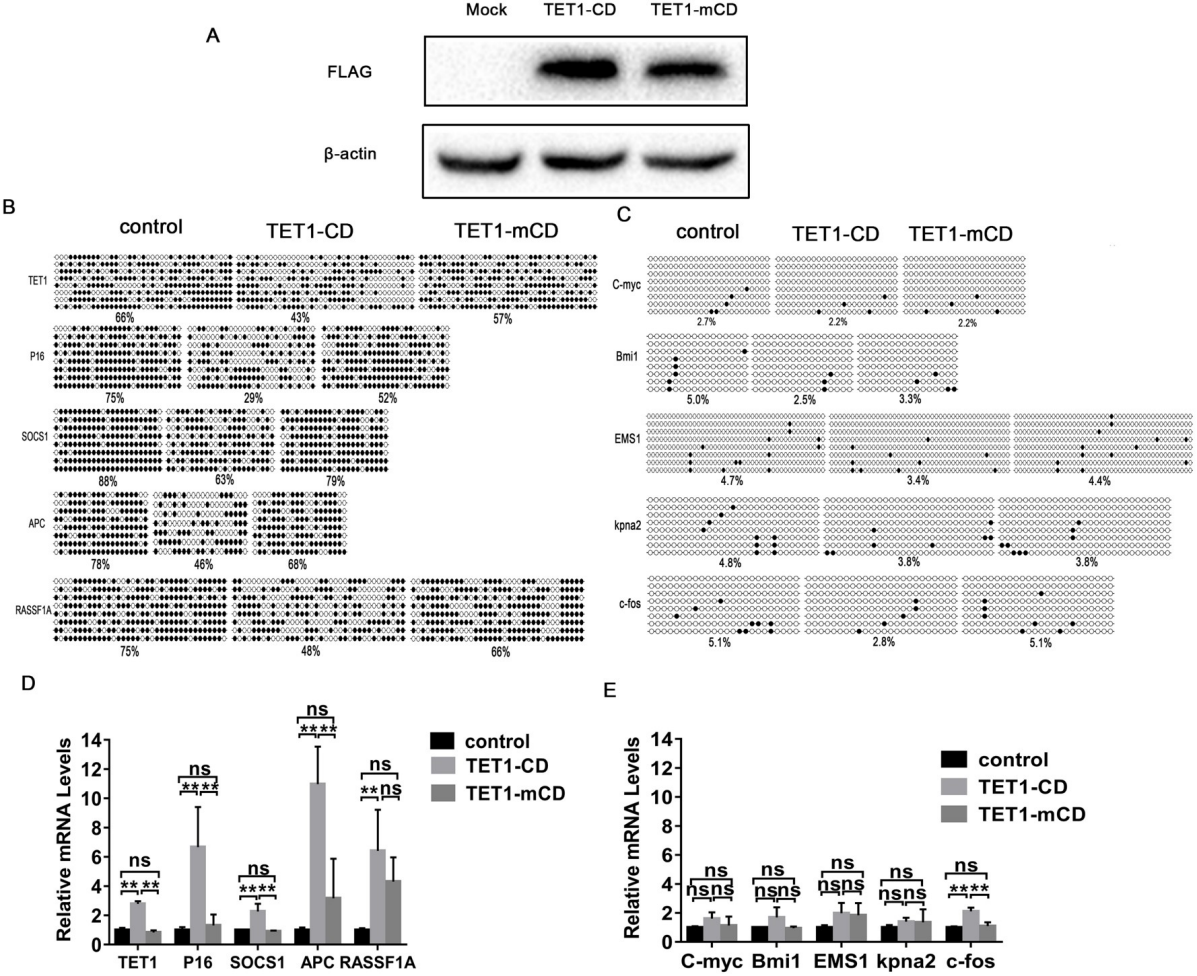
TET1-CD up-regulates TSGs expression. (A). The SMMC 7721 cells were transiently transfected with either TET1-CD plasmids or TET1-mCD plasmids. Expression of TET1-CD and TET1-mCD proteins was analysed by Western blot, and β-actin was used as an internal control. (B)&(C). The SMMC 7721 cells were cultured in the 6 mm plate for 24h and then transiently transfected with either pflag-CMV4 vector (control), TET1-CD or TET1-mCD. Expression of TSGs and oncogenes was analyzed by Quantitative RT-PCR 48h after transient transfection, and the results were represented as mean ± SD of three independent experiments. *p<0.05, **p<0.01, ***p<0.001. (one-way ANOVA).

Given that TSGs promoter CpG islands are extensively hypermethylated [9, 10] and TET1-CD induces genome-wide DNA demethylation while full length TET1 does not [26], TET1-CD was transfected into SMMC 7721 cells to verify whether it activates the hypermethylated endogenous TET1 and other TSGs. In the study, empty plasmids and TET1-CD mutants (TET1-mCD, amino acids 1672 H to Y and 1674 D to A) were both acted as control groups (Fig 2A). Bisulfite sequencing results (Fig 2B) showed that TET1-CD significantly reduced TET1, P16, SOCS1, APC and RASSF1A methylation level, while TET1-mCD did not, which is attributed to TET1-CD mutation.

Since TET1-CD can induce genome-wide DNA demethylation, it is possible that oncogenes such as C-myc, Bmi1, EMS1, Kpna2 and c-fos in hepatocellular carcinoma epigenetics [28] are also activated so as to weaken TET1-CD anticancer activity. So, the expression of oncogenes was determined. However, unexpectively, C-myc, Bmi1, EMS1, Kpna2 and c-fos demethylation levels were hardly increased by TET1-CD (Fig 2C). Compared with expression of oncogenes, expression of TSGs was drastically increased. These results suggest that TET1-CD can exert anticancer activity through its role on activation of TSGs but not through its inhibition of oncogenes. Quantitative real-time PCR results (Fig 2D and 2E) also showed that the mRNA levels of TSGs in TET1-CD group were obviously higher than that of control and p-TET1-mCD groups.

### TET1-CD inhibited SMMC 7721 cells proliferation and migration

**Fig 3:**
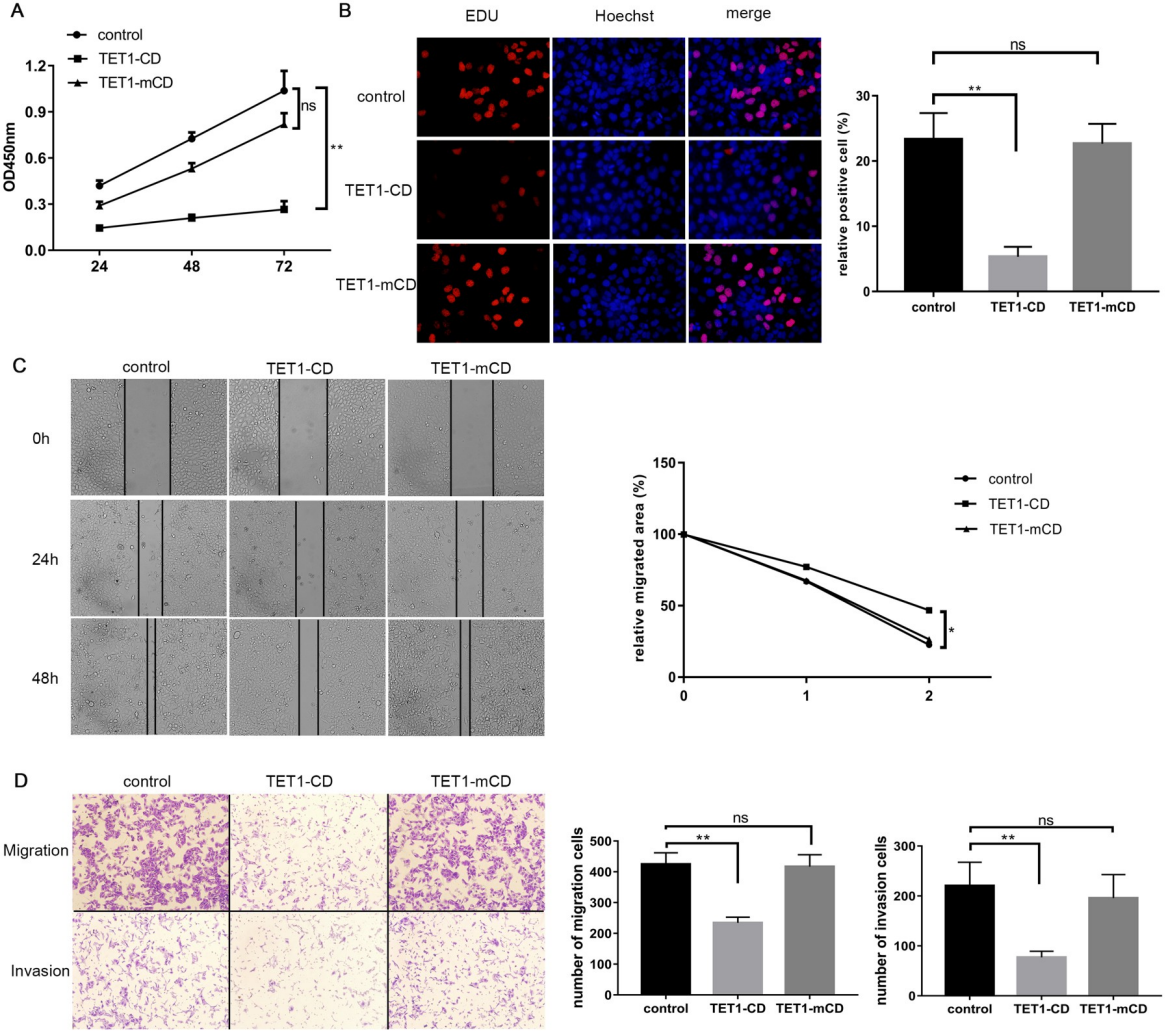
TET1-CD inhibits proliferation, migration and invasion of the SMMC 7721 cells. (A). After transfection of empty vector, TET1-CD and TET1-mCD plasmids into the SMMC 7721 cells respectively for 24h, 48h and 72h, and incubation of these cells with CCK-8 solution at 37°C for 1 h, the absorbance of these cells at 450 nm was measured to assess their proliferation. (B). The SMMC 7721 cells were inoculated into the 24-well plate, and fluorescence staining was performed 48h after inoculation. (C). Microscopy was used to capture the cell scratched area at 0h, 24h and 48h. (D). The cells, which migrated from the upper chamber to the lower chamber, were counted to assess their migration and invasion ability. The results were represented as mean ± SD of three independent experiments. One-way ANOVA, *p<0.05 and **p<0.01.

To verify TET1-CD anticancer activity, SMMC 7721 cells were transfected with TET1-CD and then cultured 24h, 48h and 72h after, respectively. Subsequently, these cells were counted, and the results (Fig 3A) showed that TET1-CD over-expression significantly inhibited proliferation of the SMMC 7721 cells over time compared with TET1-mCD and empty vector. Furthermore, EDU experiments (Fig 3B) showed TE1-CD over-expression also obviously inhibited proliferation of HCC cells.

Next, after the SMMC 7721 cells were transfected with TET1-CD and cultured for 24h and 48h, respectively, wound healing and transwell assay were utilized to evaluate the role of TET1-CD on migration and invasion of the HCC cells. Wound healing assay (Fig 3C) demonstrated that TET1-CD resulted in a higher proportion of remaining area due to its inhibiting movement of the SMMC 7721 cells to the scratched area, while TET1-mCD and empty vector gave rise to a lower proportion of remaining area due to movement of the SMMC 7721 cells to the scratched area. Again, transwell assay (Fig 3D) showed that TET1-CD distinctly repressed migration and invasion of the SMMC 7721 cells.

### TET1-CD suppressed HCC xenografts proliferation in nude mice

**Fig 4:**
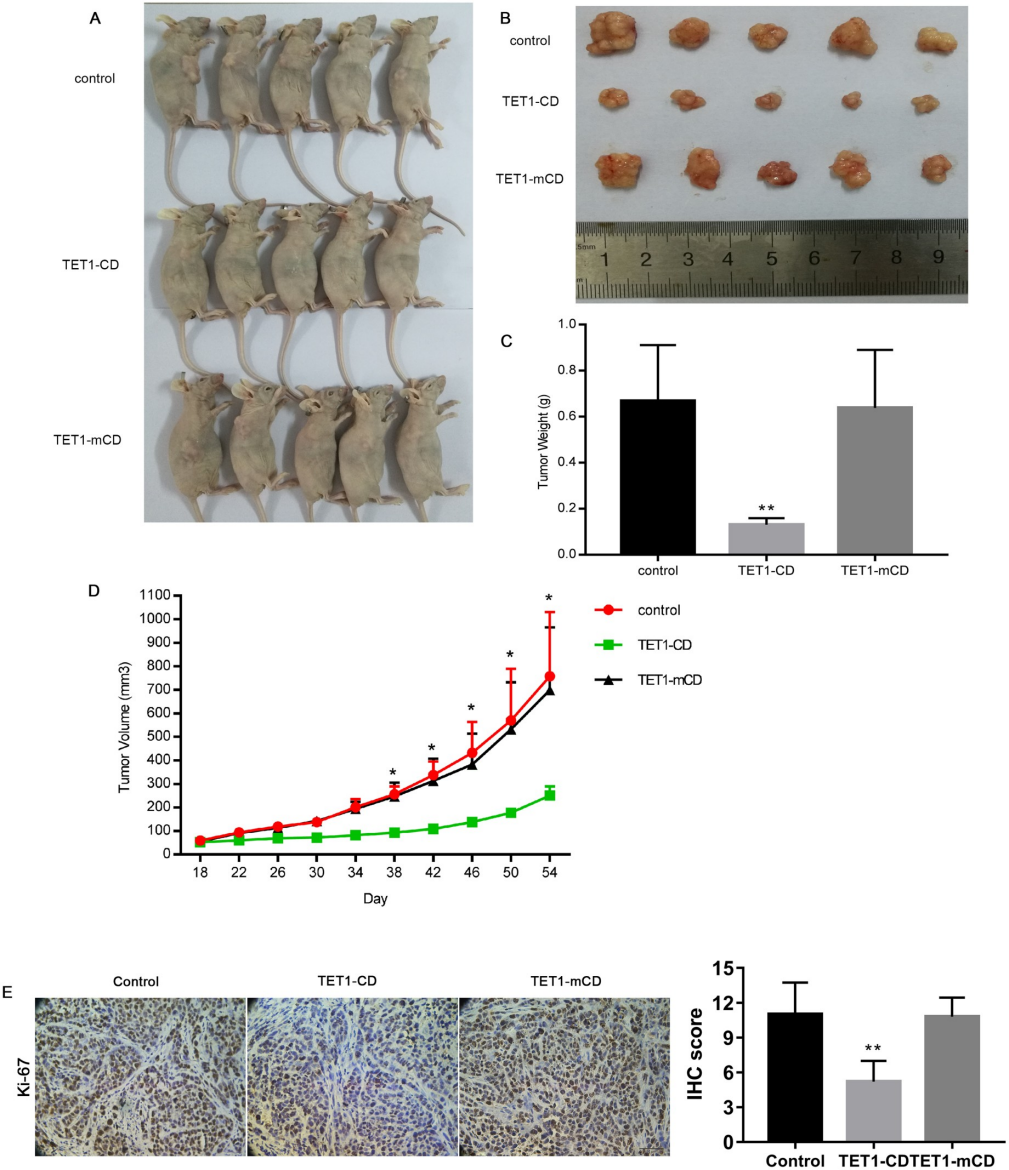
TET1-CD suppresses HCC xenografts growth in nude mice. (A). The SMMC 7721 cells, which were transiently transfected with empty vector, TET1-CD plasmids or TET1-mCD plasmids, were subcutaneously injected into the nude mice to establish a HCC subcutaneous xenograft model. The nude mice were sacrificed 54 days after the injection. (B). Photographs of excised xenograft tumors. (C). Tumor weight in the control, TET1-CD and TET1-mCD groups are shown. (D). From day 18 and 54 after the injection, the relative tumor volume was measured once every four days. The results were represented as mean ± SD (n = 5). (E). Ki-67 positive tumor cells were detected by immunohistochemistry (IHC). The results were represented as mean ± SD (n = 10). The scale bar is 50μm. One-way ANOVA, **p<0.01.

Further to confirm TET1-CD anticancer ability in vivo, TET1-CD was transiently transfected into the SMMC 7721 cells using Lipofectamine 3000, and then the transfected cells were subcutaneously injected into the nude mice to establish a HCC xenograft model. At the 54th day after a single transfection, subcutaneous tumors were taken out from the nude mice (Fig 4A and 4B), and the weight of the subcutaneous tumor taken out from the nude mice treated with TET1-CD was obviously lighter than that from the nude mice treated with TET1-mCD and empty vector (Fig 4C). From the 38th day after a single transfection on, the size of the subcutaneous tumor from the nude mice treated with TET1-CD was remarkably smaller than that from the nude mice treated with TET1-mCD and empty vector (Fig 4D).

In addition, the Ki-67 expression was assayed to assess the role of TET1-CD on proliferation of the HCC cells. The number of Ki-67 positive cells in the TET1-CD group was significantly fewer than that in the PBS and TET1-mCD groups (Fig 4E), indicating that TET1-CD suppressed proliferation of the HCC cells. These results demonstrated that TET1-CD could effectively suppressed proliferation of the HCC cells in vivo and its anticancer effects could last for a long time in spite of a single transient transfection of TET1-CD.

## Discussion

Multi-drug resistance and liver toxicity of systemic chemotherapy have long been being the main barriers that limit application of chemotherapy in HCC therapy. In the current study, we have demonstrated that a single transient transfection of TET1-CD activated multiple tumor suppressor genes, suppressed proliferation, migration and invasion of the HCC cells in vitro, and persistently repressed proliferation of HCC cells in vivo. The findings in the study have provided pathophysiological basis for clinical application of transient transfection of TET1-CD in treatment of HCC.

TET1 is known as a tumor suppressor protein although it presents carcinogenic activity in acute myeloid leukemia and gastric carcinoma [29, 30]. TET1 is not only a dioxygenase, which activates hypermethylated TSGs by demethylation, but also a DNMT3B inhibitor, which safeguards bivalent promoters from de novo methylation to ensure robust lineage-specific transcription upon differentiation [31]. TET1 deficiency not only causes cell genomic instability, a hallmark of cancer [32], but also leads to tumorigenesis and tumor vascular invasion [33, 34]. Coversely, TET1 overexpression reverses the epithelial-mesenchymal transition and inhibits cancer cell metastasis by potent inhibition of canonical Wnt/β-catenin signaling [35]. Previous studies confirmed that expression of endogenous TET1 was downregulated in HCC cells [22–24]. In the current study, likewise, decreased endogenous TET1 expression was verified in the SMMC 7721 cells, and so TET1-CD was transiently transfected into the SMMC 7721 cells to activate endogenous TET1 gene, in which its promoter CpG islands is likely hypermethylated [25], to treat endogenous TET1 deficiency, suppress proliferation, migration and invasion of the HCC cells, and promote its apoptosis.

Full length TET1 includes a catalytic center and a zinc finger domain. The catalytic center consists of a catalytic domain (CD) binding alpha ketone glutaric acid and Fe^2+^, and a cysteine-rich domain, which targets the hypermethylated CpG islands and makes be demethylated. The zinc finger domain, namely Cys-Xaa-Xaa-Cys(CXXC)close to N-terminal of TET1 protein, not only identifies and binds to the promoter, where unmethylated CpG islands frequently appear, to prevent CpG islands from being methylated by DNMTs, but also binds to the promoter, where CpG islands are hypermethylated but infrequently appear, to block further methylation [36, 37]. Thus, full length TET1 is difficult to activate a great majority of TSGs due to selective binding of CXXC, whereas TET1-CD, without the CXXC part, can activate a great majority of TSGs. Hence, TET1-CD, but not full length TET1, was transfected into the SMMC 7721 cells, and the results showed that the methylation levels of TSGs, such as TET1, P16, SOCS1, RASSF1A and APC, were significantly reduced, which are consistent with the previous findings that TET1-CD induced genome-wide DNA demethylation while full length TET1 did not [26]. In contrast, the methylation levels of oncogenes, such as C-myc, Bmi1, EMS1, Kpna2 and c-fos, were not significantly reduced. Hardly elevated expression of C-myc and c-fos might be due to hypomethylated CpG islands in their promoters and difficulties in demethylation further [38, 39], but it remains to be investigated whether hardly elevated expression of Bmi1, EMS1 and Kpna2 is also related to hypomethylation of their promoter CpG islands. So, TET1-CD possesses anticancer effects through activation of multiple TSGs but not through inhibition of oncogenes.

After hypermethylated TSGs were activated by TET1-CD, they exerted effective anticancer role (Fig 3) through multiple pathways, namely APC, p16, RASSF1A, SOCS1 and TET1. APC gene is a negative regulatory element of Wnt/β-Catenin signaling pathway[40], which plays a critical role in cancer cell proliferation and migration [41–44], and APC down-regulation or mutation promotes cell proliferation [45–47]. Efficient p16 delays cell cycle transition of G1 to S through its negative regulation of CDK4 [48, 49], and conversely deficient p16 accelerates the transition of G1 to S and leads to cell malignant proliferation as well as tumorigenicity [35, 50–52]. RASSF1A is a key member of Hippo pathway, dominantly regulating cell growth, differentiation and apoptosis, and RASSF1A over-expression significantly inhibits cell proliferation and induces a G0-G1 arrest and apoptosis [53, 54], whereas RASSF1A homozygous knockout mice develop liver tumors [55]. SOCS1is critically involved in the regulation of cellular proliferation, survival, and apoptosis via cytokine-induced JAK/STAT signaling, and defective SOCS-1 triggers JAK/STAT pathway to promote tumorigenicity [56, 57]. TET1, as discussed above, possesses anticancer activity, and conversely, TET1 downregulation or knockdown causes increased cell proliferation and migration [58]. Collectedly, TET1-CD plays an integrated anticancer role through acting on APC, p16, RASSF1A, SOCS1 and TET1 pathway. Therefore, we next investigated the effect of TET1-CD on proliferation, migration, and invasion of the SMMC 7721 cells. The CCK8 proliferation experiments showed that the absorbance at 450 nm of the p-TET1-CD group increased slowly, which was significantly lower than the absorbance of the control and p-TET1-mCD groups, which was consistent with the CCK8 experiment. Further, the scratch assay, which tests exercise capacity of the SMMC 7721 cells, showed that the scratch area of the control and p-TET1-mCD groups had obvious healing tendency after 48 hours, but the scratch area of the p-TET1-CD group was still obvious. Cell migration and invasion showed the number of cell migration and invasion in the p-TET1-CD group was significantly lower than that in the control and p-TET1-mCD groups. These indicate that TET1-CD significantly inhibits the growth and metastasis of the SMMC 7721 cells.

Strikingly, 54 days after a single transient transfection of TET1-CD, HCC subcutaneous xenografts in the nude mice remained to be significantly repressed, being likely ascribed to that endogenous TET1 gene was recurrently activated although TET1-CD expression lasts for several days at most. In the study, endogenous TET1 gene was activated after TET1-CD protein expression, and the activated endogenous TET1 gene subsequently activated the remaining endogenous TET1 gene that was still hypermethylated, which suggests that a single TET1-CD transfection actually exerts lasting anticancer effects through recurrent activation of endogenous TET1.

In summary, TET1-CD can exert integrated anticancer effects through activation of hypermethylated APC, p16, RASSF1A, SOCS1 and TET1 gene, and a single transient transfection of TET1-CD may open a new possibility for HCC treatment.

## Supporting information

S1 Table The names and sequences of PCR primers (be cited as supporting information). (DOCX)

S2 Table (DOCX)

S3 Table (DOCX)

S1 File (DOC)

S2 File (DOCX)

S3 File (DOCX)
