# Supplementary material for: Effects of a single transient transfection of Ten-eleven translocation 1 catalytic domain on hepatocellular carcinoma
Source: PLoS One. 2018 Dec 14;13(12):e0207139. doi: 10.1371/journal.pone.0207139 (PMC6294611; doi:10.1371/journal.pone.0207139)
Supplement: S2 Table — (DOCX) [file pone.0207139.s002.docx]

**S2 Table: metrological properties of techniques**

|  | **Measure** | **Muscle** | **Concurrent validity** | | **Intra rater reliability** | **Inter rater reliability** | **Other** | | **Duration** | |
| --- | --- | --- | --- | --- | --- | --- | --- | --- | --- | --- |
| **Albracht 2008 [52]** | single slice manual segmentation (CSAmax), muscle length (ML) and shape factor (p), volume: p* CSAmax* ML | GM | RMSE: 7% | | - | - | - | |  | |
|  |  | GL | RMSE: 10% | | - | - | - | |  | |
|  |  | SO | RMSE: 5% | | - | - | - | |  | |
|  |  |  |  | |  |  |  | |  | |
| **Amabile 2016 [53]** | use of ACSAmax and muscle length (ML) obtained using full muscle reconstruction and shape factor (p), volume: p* ACSAmax* ML | QL | volume RMSE: 17.7% | | - | - | - | | - | |
|  |  | ES | volume RMSE: 5.2% | | - | - | - | | - | |
|  |  | GlMa | volume RMSE: 5.9% | | - | - | - | | - | |
|  |  | GlMe | volume RMSE: 6.6% | | - | - | - | | - | |
|  |  | GlMi | volume RMSE: 11.9% | | - | - | - | | - | |
|  |  | Add OP | volume RMSE: 7.1% | | - | - | - | | - | |
|  |  | VLI | volume RMSE: 4.8% | | - | - | - | | - | |
|  |  | VM | volume RMSE: 5.2% | | - | - | - | | - | |
|  |  | TFL | volume RMSE: 9.0% | | - | - | - | | - | |
|  |  | RF | volume RMSE: 4.6% | | - | - | - | | - | |
|  |  | Gra | volume RMSE: 5.0% | | - | - | - | | - | |
|  |  | Sar | volume RMSE: 4.7% | | - | - | - | | - | |
|  |  | BFS | volume RMSE: 8.8% | | - | - | - | | - | |
|  |  | BFL | volume RMSE: 7.1% | | - | - | - | | - | |
|  |  | SM | volume RMSE: 6.7% | | - | - | - | | - | |
|  |  | ST | volume RMSE: 7.4% | | - | - | - | | - | |
|  | reduced MRI set method: model using the DPSO method, with 5 segmented slices, volume predicted from a multilinear regression | Spine flexors (iliacus, psoas) | volume RMSE: 5.7% | | - | - | - | | - | |
|  |  | spine extensors (ES+QL) | volume RMSE: 10.7% | | - | - | - | | - | |
|  |  | hip flexors (Add+Gra+Il+Ps+RF+Sar+TFL) | volume RMSE: 9.7% | | - | - | - | | - | |
|  |  | hip extensors (BFL+ BFS+GlMa+SM+ST | volume RMSE: 8.7% | | - | - | - | | - | |
|  |  | knee flexors (BFS + BFL+ Gra+Sar+SM+ST) | volume RMSE: 6% | | - | - | - | | - | |
|  |  | knee extensors (RF+ VLI+ VM) | volume RMSE: 6% | | - | - | - | | - | |
|  |  |  |  | |  |  |  | |  | |
| **Andrews 2015 [65]** | interactive segmentation using shape priors + statistical shape model | Gra | mean Surf D: 1.54; SD:0.67mm | DSI: 0.72; SD:0.24 | - | - | - | | 50+/-4.3minutes per image to run | |
|  |  | Sar |  | DSI: 0.71; SD:0.27 | - | - | - | |  |  |
|  |  | BFL |  | DSI: 0.70; SD:0.16 | - | - | - | |  |  |
|  |  | RF |  | DSI: 0.75; SD: 0.20 | - | - | - | |  |  |
|  |  | ST |  | DSI: 0.80; SD:0.16 | - | - | - | |  |  |
|  |  | BFS |  | DSI:0.89; SD:0.08 | - | - | - | |  |  |
|  |  | SM |  | DSI: 0.85; SD:0.11 | - | - | - | |  |  |
|  |  | VI |  | DSI: 0.79; SD:0.06 | - | - | - | |  |  |
|  |  | VM |  | DSI: 0.93; SD:0.06 | - | - | - | |  |  |
|  |  | Add |  | DSI: 0.81; SD:0.11 | - | - | - | |  |  |
|  |  | VL |  | DSI: 0.86; SD:0.03 | - | - | - | |  |  |
|  |  |  |  |  |  |  |  |  | |  |
| **Barnouin 2014 [46]** | slice-by-slice manual segmentation, volume: muscle tissue area * interslice distance | RF | - | | - | ICC: 0.995 significant inter rater difference mean diff: -3.8%; SD: 2.9% | contribution of individual muscles within QF significant interoperator differences for VI and VL, mean diff: 1.8% for VI; 1.3% for VL ICC 0.836-0.995 | | 5 hours/subject (whole segmentation procedure) | |
|  |  | VI | - | | - | ICC:0.997 no significant inter rater difference mean diff: -1.4%; SD: 3.3% |  |  |  |  |
|  |  | VL | - | | - | ICC: 0.988 significant inter rater difference mean diff: -4.5%; SD: 2.9% |  |  |  |  |
|  |  | VM | - | | - | ICC: 0.992 significant inter rater difference mean diff: -3.1%; SD: 1.9% |  |  |  |  |
|  |  | Qua | - | | - | ICC: 0.995 significant inter rater difference mean diff: -3.1%; SD: 1.8% |  |  |  |  |
|  |  |  |  | |  |  |  | |  | |
| **Barnouin 2015 [47]** | slice-by-slice manual segmentation, volume: cylinder/ cone method/ 3d-order polynomial regression/ 4th-order polynomial regression | RF, VI, VL, VM | effect of the method statistically significant for all the muscles. mean diff < 1% ( all individuals and muscles) | | - | - | mean CV between methods= 0.41% (SD=0.45%, range: 0.03–2.75%) | | - | |
|  | manual segmentation of a reduced number of slices, volume: cylinder/ cone method/ 3d-order polynomial regression/ 4th-order polynomial regression |  | inter-slice distances >5 cm: none of the methods correctly estimate the muscle volume (error> 5%). increasing the distance between slices: greater inter-individual variability | | - | - | effect of inter-slice distance very significant for the TC method and the VM muscle. most stable method: cylinder method | | - | |
|  |  |  |  | |  |  |  | |  | |
| **Belavy 2011 [55]** | manual segmentation of a reduced number of slices, selection of the segmented slices with 5 algorithms including subalgorithms with various number of slices (1-largest CSA and the sum of the 3,6,9 … largest CSA measurement/ 2-largest CSA with immediately adjacent CSAs/ 3-same as 2 except every second images taken/ 4- method using CSA at 30, 40, 50, 80%/ 5- most psoximal CSA with every 2d, 3d, 4th... CSA measurements), volume: linear interpolation | RF, VM, VL, VI, Sar, Gra,Add M, Add L, BFL, BFS,ST, SM, GL, GM, SO+FHL, TP, FDL, PER LBT, TA +EDL + EHL, left side | As the number of slices included in the measure of muscle size increased, the correlation with muscle volume tended to approach 1. All the correlations were statistically significant. 366 individual CSA measurements for a typical data set (measurements from all of the muscles on one lower limb) Algorithms 1, 2 or 3, required 193, 191 or 232 individual CSA measurements to accurately estimate the volumes of all lower-limb muscles considered. Depending on the muscles, algorithms and subalgorithms differed. | | - | - | - | | - | |
|  |  |  |  |  |  |  |  |  |  |  |
|  |  |  |  | |  |  |  | |  | |
| **Elliott, 1997 [66]** | image based segmentation + manual segmentation correction algorithm for partial volume effect, volume: addition of the number of voxels | GM, GL, So | - | | - | for 9 muscles (3 GM, 3GL, 3So) correlation coefficient: 0.99 max diff: 4.3% | - | | average time to segment an individual muscle: 45 minutes. | |
|  |  |  |  |  |  |  |  |  | |  |
| **Eng 2007 [54]** | manual segmentation in the 3 planes, volume: addition of the number of voxels | PT | ICC: 0.97 mean diff: 8.4% | | - | ICC: 0.97 mean diff: 8.8% | *-* | | - | |
|  |  | ECRB | ICC: 0.93 mean diff: 7.7% | | - |  | *-* | | - | |
|  |  | EPL | ICC: 0.68 mean diff: 21.6% | | - |  | *-* | | - | |
|  |  | FCU | ICC: 0.91 mean diff: 9.8% | | - |  | *-* | | - | |
|  |  | BR | ICC: 0.93 mean diff: 17.2% | | - |  | *-* | | - | |
|  |  |  |  | |  |  |  | |  | |
| **Engstrom 2011 [67]** | atlas based + statistical shape based segmentation | QL | DSI: 0.86; SD:0.06 TC: 0.75; SD: 0.08 mean Surf D: 1.32mm; SD:0.60mm | | - | - | results errors obtained from non rigid registration and 3D SSM segmentation methods alone were greater than atlas based + statistical shape based segmentation method | | - | |
|  |  | Ps | DSI: 0.91; SD:0.03 TC: 0.84; SD:0.05 mean Surf D: 1.42mm; SD:0.51mm | | - | - |  |  | - | |
|  |  | ESM | DSI: 0.92; SD:0.03 TC: 0.85; SD:0.06 mean Surf D: 1.81mm; SD:0.81mm | | - | - |  |  | - | |
|  |  |  |  |  |  |  |  |  | |  |
| **Jolivet 2014 [68]** | improved DPSO | RF, VLMI, Sar, TFL, BFS, BFL, ST, SM, Gra | volume 6 manual contours required for ST muscle, 5 for BFS, RF and SAR and 4 for BFL,GRA, SM, TFL and VLMI, instead of 7 for RF and SAR, 6 for BFL, 5 for BFS, GRA, SM, TFL and ST, 4 for VLMI with the non-optimised method. point to surface distance 5 manual contours required for RF, SM, ST and VLMI muscles; 4 for BFS, BFL, SAR and TFL; 3 for GRA instead of 7 for Sar; 6 for BFL, RF, Gra, SM, ST, VLMI, 5 for BFS; 4 for TFL | | - | - | - | | - | |
|  |  |  |  | |  |  |  | |  | |
| **Kim 2017 [29]** | thresholding and manual post- processing | Sspi | - | | - | DSI: 0.95 (SD: 0.012) kappa: 0.948 (SD:0.012) | - | | - | |
|  | image based and shape based segmentation |  | DSI: 0.95 accuracy: 0.99 mean Surf D: 0.44mm max Surf D: 3.04mm | | - | - | - | | - | |
|  |  |  |  |  |  |  |  |  | |  |
| **Lehtinen 2003 [56]** | single slice manual segmentation (at the Y-shaped position), volume: calculated by the software | Sspi | mean volume: 27cm^3^ (ref volume: 36cm^3^); 2SD: 18cm^3^ | | CV: 4.2% | CV: 1.6% | - | | average time to complete the tracing of the 3 muscles: 70sec (30min for slice-by-slice segmentation) | |
|  |  | Ssca | mean volume: 80cm^3^ (ref volume: 99cm3); 2SD: 50cm^3^ | | CV: 4.5% | CV: 4.4% | - | |  |  |
|  |  | Ispi+Tmin | mean volume: 87cm^3^ (ref volume: 96cm^3^); 2SD: 72cm^3^ | | CV: 3.0% | CV: 2.7% | - | |  |  |
|  | manual segmentation of 2 slices (at the Y-shaped position and at a defined more medial position), volume: calculated by the software | Sspi | mean volume: 32cm^3^ (ref volume: 36cm^3^); 2SD: 20cm^3^ | | CV: 3.3% | CV: 1.6% | - | | average time to complete the tracing of the 3 muscles: 115sec (30min for slice-by-slice segmentation) | |
|  |  | Ssca | mean volume: 91cm^3^ (ref volume: 99cm3); 2SD: 61cm^3^ | | CV: 3.5% | CV: 2.6% | - | |  |  |
|  |  | Ispi+Tmin | mean volume: 89cm^3^ (ref volume: 96cm^3^); 2SD: 79cm^3^ | | CV: 2.5% | CV: 1.0% | - | |  |  |
|  |  |  |  | |  |  |  | |  | |
| **Le Troter 2016 [48]** | slice-by-slice manual segmentation, volume: cone method | RF | - | | - | - | Interscan reliabilityy: ICC>= 0.98 for all the muscles | volume CV: 4.5% | | - |
|  |  | VI | - | | - | - |  | volume CV: 2.1% | | - |
|  |  | VL | - | | - | - |  | volume CV: 5.5% | | - |
|  |  | VM | - | | - | - |  | volume CV: 1.7% | | - |
|  |  | Qua | - | | - | - |  | volume CV: 2% | | - |
|  | atlas based segmentation (semi automated) | RF | volume ICC: 0.99; CV: 3.6% DSI: 0.89; SD:0.07 FNVF: 0.10; SD:0.06 FPVF: 0.10; SD:0.09 MVSF: 0.05; SD:0.09 | | - | - | - | | - | |
|  |  | VI | volume ICC: 0.98; CV: 1.1% DSI: 00.94; SD:0.01 FNVF: 0.05; SD:0.02 FPVF: 0.05; SD:0.02 MVSF: 0.02; SD:0.02 | | - | - | - | | - | |
|  |  | VL | volume ICC: 0.99; CV: 2.1% DSI: 0.93; SD: 0.03 FNVF: 0.07; SD: 0.03; FPVF: 0.07; SD: 0.05 MVSF: 0.03; SD: 0.04 | | - | - | - | | - | |
|  |  | VM | volume ICC: 0.98; CV: 1.8% DSI: 0.95; SD: 0.04 FNVF: 0.06; SD:0.05 FPVF: 0.04; SD:0.04 MVSF: 0.02; SD:0.03 | | - | - | - | | - | |
|  | atlas based segmentation (fully automated) | RF | volume ICC: 0.78; CV: 17.3% DSI: 0.84; SD:0.12 FNVF: 0.20; SD:0.24 FPVF: 0.33; SD:0.13 MVSF: 0.23; SD:0.24 | | - | - | best results obtained with the combination of 2 deformation fields resulting from the non-parametric symmetric diffeomorphic normalization and the STEPS fusion algorythm, | | - | |
|  |  | VI | volume ICC: 0.70; CV: 10.5% DSI: 0.87; SD:0.07 FNVF: 0.12; SD:0.11 FPVF: 0.13; SD:0.05 MVSF: 0.10; SD:0.07 | | - | - |  |  | - | |
|  |  | VL | volume ICC: 0.90; CV: 17.3% DSI: 0.88; SD:0.08 FNVF: 0.05; SD:0.05 FPVF: 0.23; SD:0.16 MVSF: 0.23; SD:0.23 | | - | - |  |  | - | |
|  |  | VM | volume ICC: 0.96; CV: 3.9% DSI: 0.91; SD:0.05 FNVF: 0.10; SD:0.06 FPVF: 0.08; SD:0.06 MVSF: 0.04; SD:0.04 | | - | - |  |  | - | |
|  |  |  |  |  |  |  |  |  | |  |
| **Lund 2002 [49]** | manual segmentation of a reduced number of slices (13 slices) | TA+EDL+EHL, left side | - | | ICC: 0.99 significant difference mean diff: 0.07% | ICC: 0.96 significant difference mean diff: 4.7% | - no difference between cone and cylinder method using 50 slices - mean difference: 0.31% between cone and cylinder method using 8 slices | | - | |
|  | slice-by-slice manual segmentation, volume: cylinder/ cone method |  | mean diff: 0.31ml, 2SD: 0.66ml | | - | - |  |  | - | |
|  | manual segmentation of a reduced number of slices (8 slices), volume: cylinder method |  | mean diff: 0.3ml (0.004%); 2SD: -0.66ml | | - | - |  |  | - | |
|  | manual segmentation of a reduced number of slices (8 slices), volume: cone method |  | - | | - | - |  |  | - | |
|  |  |  |  |  |  |  |  |  | |  |
| **Marcon 2015 [9]** | manual segmentation of a reduced number of slices (every third slice), volume: NR | Qua | - | | ICC: 0.90 | - | - |  | | - |
|  | single slice manual segmentation (at 25cm above the knee joint), volume: NR | Qua | SEE: 8.1% |  | - | - | - |  | | - |
|  |  |  |  | |  |  |  | |  | |
| **Mersmann 2014 [57]** | single slice manual segmentation (CSA max), muscle length (ML) and shape factor (p), volume: p* ACSAmax* ML | SO | r^2^: 0.864 no significant difference volume RMSE: 7.9% | | - | - | - | | - | |
|  |  | GM | r^2^: 0.953 no significant difference volume RMSE: 4.8% | | - | - | - | | - | |
|  |  | GL | r^2^: 0.849 no significant difference volume RMSE: 8.3% | | - | - | - | | - | |
|  |  |  |  | |  |  |  | |  | |
| **Mersmann 2015 [58]** | single slice manual segmentation (CSA max), muscle length (ML), and shape factor (p), volume: p* ACSAmax* ML | VI | r^2^: 0.955 no significant difference volume RMSE: 5.2% | | - | - | - | | - | |
|  |  | VL | r^2^: 0.972 no significant difference volume RMSE: 4.6% | | - | - | - | | - | |
|  |  | VM | r^2^: 0.943 no significant difference volume RMSE: 5.7% | | - | - | - | | - | |
|  |  |  |  | |  |  |  | |  | |
| **Moal 2014 [59]** | DPSO method T1 images | Add BLM, BF, ES, GlMa, GlMe, GlMi, Gra, Il, Obl, Ps, QL, RA, RF, Sar, SMT, TFL, VLI, VM | on all the muscles, mean diff: 1.10%; SD: 2.50%; mean point to surface distance 2RMSE< 3mm, (0.88mm-11.30mm) | | on all the muscles, mean CV: 2.16%; SD: 0.86%; range: 0.95 - 3.75% GlMi: CV> 5% | on all the muscles, mean CV: 2.55%; SD: 1.07%; range: 0.95- 5.78% GlMi, GlMe, RA: CV> 5% | no significant differences between sequences for intra and inter rater reliability | | time to obtain a reconstruction reference method: 14-15 hours time to obtain a reconstruction DPSO method: 7 hours | |
|  | DPSO method Fat images |  | on all the muscles, mean diff: 2.19%; SD: 2.85%; mean point to surface distance 2RMSE< 3mm, (1.15mm-16.41mm) | | on all the muscles, mean CV: 2.05%; SD: 1.01%; range: 0.90 - 5.62% GlMi: CV> 5% | on all the muscles, mean CV: 2.61%; SD: 1.82%; range: 0.93 - 11.80% GlMi, GlMe, RA: CV> 5% |  |  |  |  |
|  |  |  |  |  |  |  |  |  | |  |
| **Morse 2007 [60]** | single slice manual segmentation (CSAmax), volume: equation using length and CSA max | VL | r^2^: 0.726 | | - | - | - | | - | |
|  |  | VI | r^2^: 0.810 | | - | - | - | | - | |
|  |  | VM | r^2^: 0.798 | | - | - | - | | - | |
|  |  | RF | r^2^: 0.694 | | - | - | - | | - | |
|  |  | Qua | r^2^: 0.945 SEE: 4.5%; SD: 2.7%, mean diff: 25.6 cm^3^; 1.96SD: 250 cm^3^ | | - | - | - | | - | |
|  | single slice manual segmentation (CSA at 40% from the distal end of the femur), regression equation to estimate the maximum muscle cross-sectional area, volume: equation using length and CSA max | Qua | r^2:^: 0.84 SEE: 26.8%; SD: 5.2% mean diff: 551 cm^3^; 1.96SD: 141 cm^3^ | | - | - | - | | - | |
|  | single slice manual segmentation (CSA at 50% from the distal end of the femur), regression equation to estimate the maximum muscle cross-sectional area, volume: equation using length and ACSA max | Qua | r^2^: 0.93 SEE : 12.5%; SD: 5.4% mean diff: 254cm^3^; 1.96SD: 107 cm^3^ | | - | - | - | | - | |
|  | single slice manual segmentation (CSA at 60% from the distal end of the femur), regression equation to estimate the maximum muscle cross-sectional area, volume: equation using length and ACSA max | Qua | r^2^: 0.90 SEE: 9.9%; SD: 5.7% mean diff: –190 cm^3^; 1.96SD: 135 cm^3^ | | - | - | - | | - | |
|  |  |  |  | |  |  |  | |  | |
| **Nordez 2009 [27]** | slice-by-slice manual segmentation,volume using 3D shape | Qua (VL+VI+VM+RF) |  | | ICC: 0.999 CV: 0.5% | ICC: 0.997 CV: 0.8% | global reliability: 1.1% | | - | |
|  | manual segmentation of a reduced number of slices, volume: cone method |  | volume error decreased when the number of available slices increased for VL+VI, VM, RF, quadriceps | with 12 slices, mean diff: 0.7%; 1.96SD: 0.7% | - | - | cone method error higher than that of the Cavalieri and DPSO methods from 3 to 11 slices. Cavalieri method error higher than that of the DPSO method from 3 to 7 slices. Number of slices required to reach a given volume error different for the different methods. | | - | |
|  | manual segmentation of a reduced number of slices, volume: Cavalieri formula |  |  | with 9 slices mean diff: 0.7%; 1.96SD: 0.9% | - | - |  |  | - | |
|  | manual segmentation of a reduced number of slices, cubic spline interpolation to estimate missing CSAs |  |  | with 5 slices mean diff: 1.0%; 1.96SD: 2.3% | - | - |  |  | - | |
|  | manual segmentation of a reduced number of slices, volume: DPSO |  |  | with 7 slices mean diff: 0.9%; 1.96SD: 1.1% | - | - |  |  | - | |
|  |  |  |  |  |  |  |  |  | |  |
| **Popadic 2011 [50]** | single slice manual segmentation (CSA max), humerus length (HL) volume: equation using CSA max, humerus length (HL), BMI | TB | mean volume change before/after training using multi slice calculation: +7.8% / using model A: +6.3% / using model CSAmax: +6.4% / using model CSA50%: +3.3% / using model CSA60%: +7% | volume r^2^: 0.830 RSE: 8.0% | - | - | - | | - | |
|  | single slice manual segmentation (CSA max), humerus length (HL) volume: equation using CSA max, HL |  |  | volume r^2^: 0.805 RSE: 8.55% | - | - | - | | - | |
|  | single slice manual segmentation (CSA 50%), humerus length (HL) volume: equation using CSA50%, HL |  |  | volume r^2^: 0.842 RSE=7.7% | - | - | - | | - | |
|  | single slice manual segmentation (CSA 60%), humerus length (HL) volume: equation using CSA60%, HL |  |  | volume r^2:^ 0.836 RSE:7.8% | - | - | - | | - | |
|  |  |  |  | |  |  |  | |  | |
| **Skorupska 2016 [61]** | slice-by-slice manual segmentation, volume: addition of the voxels and multiplication by the voxel dimension | Pir | - | | - | healthy: ICC: 0.94 low back pain: ICC: 0.97 | - | | - | |
|  |  | GlMi | - | |  | healthy: ICC: 0.99 low back pain: ICC: 0.98 | - | | - | |
|  |  | GlMe | - | | - | healthy: ICC: 0.984 low back pain: ICC: 0.848 | - | | - | |
|  |  | GlMa | - | | - | healthy: ICC: 0.942 low back pain: ICC: 0.969 | - | | - | |
|  |  |  |  | |  |  |  | |  | |
| **Smeulders 2010 [62]** | slice-by-slice manual segmentation, volume: muscle tissue area * interslice distance | FCU | - | | ICC:1.0 no significant difference mean diff: 0.21ml; SD: 0.26ml; CV: 0.8% | ICC: 0.99 no significant difference mean diff: 0.45ml; SD: 1.02ml; CV: 3.3% | Interscan reliability: ICC: 0.99 no significant difference mean diff: 0.27ml; SD: 1.11ml; CV: 3.6% SDD: 2.2 mL | | - | |
|  |  | ECU | - | | ICC:1.0 no significant difference mean diff: -0.08ml; SD: 0.30ml; CV: 1.8% | ICC: 0.99 significant difference mean diff: -0.87ml; SD: 0.94ml; CV: 5.7% | Interscan reliability: ICC: 0.99 no significant difference mean diff: -0.34ml; SD: 0.50 ml; CV: 3.0% SDD: 1.0 mL | | - | |
|  |  |  |  | |  |  |  | |  | |
| **Springer 2012 [63]** | slice-by-slice manual segmentation, volume: NR | GlMe | - | | no significant difference non operated side mean diff: 0.1%; 1.96SD: 5.4%; CV: 2.0% operated side mean diff: -0.7%; 1.96SD: 5.6%; CV: 2.4% | significant difference on both sides non operated side mean diff: 6.4%; 1.96SD: 13.7%; CV: 5.2% operated side mean diff: 10.2%; 1.96SD: 10.2%; CV: 4.6% | - | | - | |
|  |  | GlMi | - | | no significant difference non operated side mean diff: 1.0%; 1.96SD: 4.3%; CV: 5.2% operated side: mean diff: -2.5%; 1.96SD : 12.2%; CV: 18.8% | significant difference on both sides non operated side mean diff: 5.9%; 1.96SD: 5.8%; CV: 5.2% operated side mean diff: 5.2%; 1.96SD: 8.0%; CV:13.9% | - | | - | |
|  |  | OE | - | | no significant difference non operated side mean diff: -0.1%; 1.96SD: 2.6%; CV: 5.7% operated side: mean diff: -0.4%; 1.96SD: 3.2%; CV: 8.4% | no significant difference non operated side mean diff: 1.7%; 1.96SD: 6.1%; CV: 14% operated side mean diff: 0.8%; 1.96SD: 5.4%; CV:15% | - | | - | |
|  |  |  |  | |  |  |  | |  | |
| **Sudhoff 2009 [64]** | slice-by-slice manual segmentation (T1 images), volume: using 3D shape | SM, ST, BFS, BFL, Sar, TFL, Gra, VLI, VM, RF, GM, GL | - | | - | For one subject: mean diff<6%, except for the BFS, Sar, Gra (10%) point to surface distance 1RMSE<1mm, 2RMSE<3mm. | - | | reconstruction of 12 muscles: 1h (12h when using manual slice-by-slice segmentation) | |
|  | DPSO | SM, 6 slices | for 2 subjects mean diff: 2.18%, point to surface distance error: 2.57mm | | ICC: 0.99 mean diff: 5.8%; 2SD: 5.4% point to surface distance 2RMSE: 2.8mm | - | - | |  |  |
|  |  | ST, 6 slices | for 2 subjects mean diff: 3.55% point to surface distance error: 3.06mm | | ICC: 0.96 mean diff: 7.5%; 2SD: 6.5% point to surface distance 2RMSE: 2.7mm | - | - | |  |  |
|  |  | BFL, 6 slices | for 2 subjects mean diff: 1.28% point to surface distance error: 1.60mm | | ICC: 0.99 mean diff: 5.9%; 2SD: 5,6% point to surface distance 2RMSE: 3.3mm | - | - | |  |  |
|  |  | BFS, 8 slices | for 2 subjects mean diff: 4.17% point to surface distance error: 2.40mm | | ICC: 0.96 mean diff: 10.8%; 2SD: 10.6% point to surface distance 2RMSE: 2.7mm | - | - | |  |  |
|  |  | SAR, 7 slices | for 2 subjects mean diff: 2.87% point to surface distance error: 3.49mm | | ICC: 0.93 mean diff: 8.4%; 2SD: 10.9% point to surface distance 2RMSE: 3.6mm | - | - | |  |  |
|  |  | TFL, 6 slices | for 2 subjects mean diff: 2.73% point to surface distance error: 1.29mm | | ICC: 0.98 mean diff: 4.3%; 2SD: 6.0% point to surface distance 2RMSE: 3.1mm | - | - | |  |  |
|  |  | GRA, 7 slices | for 2 subjects mean diff: 3.27% point to surface distance error: 2.51mm | | ICC: 0.99 mean diff: 8.6%; 2SD: 5,2% point to surface distance 2RMSE: 3.1mm | - | - | |  |  |
|  |  | VLI, 7 slices | for 2 subjects mean diff: 3.12% point to surface distance error: 4.89mm | | ICC: 0.99 mean diff: 1.9%; 2SD: 2.7% point to surface distance 2RMSE: 5.6mm | - | - | |  |  |
|  |  | VM, 7 slices | for 2 subjects mean diff: 3.35% point to surface distance error: 3.92mm | | ICC: 0.99 mean diff: 3.2%; 2SD: 3.4% point to surface distance 2RMSE: 3.7mm | - | - | |  |  |
|  |  | RF, 6 slices | for 2 subjects mean diff: 0.60% point to surface distance error: 3.60mm | | ICC: 0.98 mean diff: 4.9%; 2SD: 4.3% point to surface distance 2RMSE: 3.4mm | - | - | |  |  |
|  |  | GM, 8 slices | for 2 subjects mean diff: 3.28% point to surface distance error: 3.31mm | | ICC: 0.96 mean diff: 5.6%; 2SD: 8.9% point to surface distance 2RMSE: 6.3mm | - | - | |  |  |
|  |  | GL, 6 slices | for 2 subjects mean diff: 2.01% point to surface distance error: 2.40mm | | ICC: 0.86 mean diff:: 7.1%; 2SD: 13.9% point to surface distance 2RMSE: 6.1mm | - | - | |  |  |
|  |  |  |  |  |  |  |  |  | |  |
| **Tingart 2003 [25]** | slice-by-slice manual segmentation, volume: muscle tissue area * interslice distance | Sspi | r^2^: 0.99 mean diff: 4%; SD 3% | | r ^2^: 0.98 CV: 2.56% | r^2^: 0.97 CV: 3.63% | - | | 7 minutes | |
|  |  | Ssca | r^2^: 0.99 mean diff : 3%; SD 2% | | r ^2^: 0.98 CV: 2.05% | r^2^: 0.98 CV: 1.76% | - | | 13 minutes | |
|  |  | Ispi+Tmin | r^2^: 0.996 mean diff: 2%; SD 2% | | r ^2^: 0.98 CV: 1.94% | r^2^: 0.98 CV: 1.83% | - | | 12 minutes | |
|  |  |  |  |  |  |  |  |  | |  |
| **Tracy 2003 [26]** | manual segmentation of a reduced number of slices, muscle volume: cone method | Qua | significant underestimation of MV for each of the alternative measures, least for MV2 and greatest for MV10. significant underestimation of change of MV by method using every 8th/ 10th section | for every 4th slices (3.1cm gap) baseline MV mean diff: 23cm^3^, 2SD: 1.7% MV change | - | - | - | | 3/4 of time gained with every 4 slices segmentation | |
|  |  |  |  | mean diff: 0.25cm3, 2SD: 16.4% |  |  |  |  |  |  |
|  | single slice manual segmentation (CSAmax), volume: univariate regression |  | baseline MV r^2^: 0.96 2SEE: 14.1% MV change r^2^: 0.74 2SEE: 59.7% |  | - | - | - | | - | |
|  |  |  |  | |  |  |  | |  | |
| **Valentin 2015 [45]** | slice-by-slice manual segmentation, volume: muscle tissue area * interslice distance | RA | - | | - | ICC: 0.77 mean diff: 2%; 2SD: 3% | - | | - | |
|  |  | Ps | - | | - | ICC: 0.92 mean diff: 0.5%; 2SD: 3% | - | | - | |
|  |  | M | - | | - | ICC: 0.59 mean diff: -4%; 2SD: 5% | - | | - | |
|  |  | ES | - | | - | ICC: 0.93 mean diff: 1%; 2SD: 2% | - | | - | |
|  |  | MES | - | | - | ICC: 0.904 | - | | - | |
|  |  |  |  | |  |  |  | |  | |
| **Vanmechelen 2017 [51]** | single slice manual segmentation (CSAmax) muscle length (ML) obtained using full muscle reconstruction and form factor (FF), volume: ((CSAmax* ML)-Offset)*FF | GM | r^2^: 0.998 SEE: 5.3% |  | - | - | - |  | | - |
|  |  | SOL | r^2^: 0.993 SEE: 8.9% |  | - | - | - |  | | - |
|  |  | TA | r^2^: 0.994 SEE: 8.7% |  | - | - | - |  | | - |
|  |  | RF | r^2^: 0.988 SEE: 4.8% |  | - | - | - |  | | - |
|  |  | SM | r^2^: 0.996 SEE: 6.5% |  | - | - | - |  | | - |
|  |  | ST | r^2^: 0.994 SEE: 9.0% |  | - | - | - |  | | - |
|  |  |  |  |  |  |  |  |  | |  |
| **Yamauchi 2017 [28]** | single slice manual segmentation (CSAs at 60% of the femoral length),femoral length (FL), volume: regression equations which varied for each muscle | RF | No significant difference between measured and estimated MVs | SEE: 12.5% | - | - | - |  | | - |
|  |  | VL |  | SEE: 7.1% | - | - | - |  | | - |
|  |  | VI |  | SEE: 7.5% | - | - | - |  | | - |
|  |  | VM |  | SEE: 8.1% | - | - | - |  | | - |
|  |  | BFS |  | SEE: 14.4% | - | - | - |  | | - |
|  |  | BFL |  | SEE: 7.2% | - | - | - |  | | - |
|  |  | ST |  | SEE: 10.9% | - | - | - |  | | - |
|  |  | SM |  | SEE: 13.9% | - | - | - |  | | - |
|  | use of muscle thickness at 50% of the femur and femoral length (FL), volume: regression equations which varied for each muscle | RF | No significant difference between measured and estimated MVs | SEE: 21.8% | - | - | - |  | | - |
|  |  | VL |  | SEE: 13.0% | - | - | - |  | | - |
|  |  | VI |  | SEE: 15.5% | - | - | - |  | | - |
|  |  | VM |  | SEE: 18.6% | - | - | - |  | | - |

Table 3 : metrological properties of techniques

ICC: intraclass correlation coefficient, mean diff: mean difference, SD: standard deviation, CV: coefficient of variation, SDD: smallest detectable difference, RMSE: root mean square error, SEE: standard error of the estimate, DSI: Dice similarity index, mean surf D: mean surface distance, max surf D: maximal surface distance, TC: Tannimoto coefficient, FNVF: false negative volume fraction, FPVF: false positive volume fraction, MVSF: muscle volume similarity fraction

RF: rectus femoris, VI: vastus intermedius, VL: vastus lateralis, VM : vatsus medialis, Qua : quadriceps, Pir : Piriformis, GlMi : Gluteus Minimus, GlMe : Gluteus Medius, GlMa : Gluteus Maximus, FCU: flexor carpi ulnaris, ECU: extensor carpi ulnaris, Sspi: Supraspinatus, Ssca: Subscapularis, Ispi+Tmin: Infraspinatus and Teres minor, ES: Erector Spinae, M: multifidus, RA: rectus abdominis, Ps: Psoas, Sar: Sartorius, Gra: Gracilis, AddM: Adductor Magnus, Add L: Adductor longus, BFL: Biceps Femoris Long head, BFS: Biceps Femoris Short head, ST: Semi Tendinosus, SM: Semi Membranosus, GL: Gastrocnemius Lateralis, GM: Gastrocnemius Medialis, So+FHL: Soleus and flexor hallucis longus, TP: Tibialis Posterior, FDL: flexor digitorum longus, Per LBT: Peroneus (Longus, Brevis, Tertius), TA+EDL+EHL: tibialis anterior and extensor digitorum longus and extensor hallucis longus, So: Soleus, TS: triceps surae, TB: triceps brachii, TA: Tibialis Anterior, VLMI: Vastus Lateralis and Medius and Intermedius, TFL: tensor Fascia Lata, Add BLM: adductor (brevis, longus, magnus), Il: Iliacus , Obl: Obliquus (transversus abdominis, internus and externus obliquus), QL: Quadratus Lumborum, VLI: Vastus Lateralis and Intermedius together, VLMI: Vastus Lateralis and Medialis and Intermedius, BF: Biceps Femoris, SMT: Semi Membranosus and Tendinosis, ESM : erector spinae and multifidus, PT: pronator teres, ECRB : Extensor Carpi Radialis Brevis, EPL : Extensor Pollicis Longus, Br : Brachioradialis
