# Supplementary material for: Effects of a single transient transfection of Ten-eleven translocation 1 catalytic domain on hepatocellular carcinoma
Source: PLoS One. 2018 Dec 14;13(12):e0207139. doi: 10.1371/journal.pone.0207139 (PMC6294611; doi:10.1371/journal.pone.0207139)
Supplement: S3 Table — (DOCX) [file pone.0207139.s003.docx]

**S3 Table: validity and reliability of the articles included**

|  | **validity** | **intra rater reliability** | **intra rater reliability** | **comparison between methods** |
| --- | --- | --- | --- | --- |
| **slice-by-slice CSA segmentation** | Tingart 2003 [25], | Tingart 2003 [25], Smeulders 2010 [62], Springer 2012 [63], Nordez 2009 [27] | Tingart 2003[25], Smeulders 2010 [62], Springer 2012 [63], Barnouin 2014 [46], Valentin 2015 [45], Skorupska 2016 [61], Sudhoff 2009 [64], Nordez 2009 [27] | Lund 2002 [49] |
| **segmentation of CSA in a reduced number of slice[s]** | Nordez 2009 [27], Tracy 2003 [26], Barnouin 2015 [47], Lund 2002 [49], Belavy 2011 [55] | Marcon 2015 [9], Lund 2002 [49] | Lund 2002 [49] |  |
| **using CSA segmentation on a single slice and muscle length** | Albracht 2008 [52], Amabile 2016 [53], Mersmann 2014 [57], Mersmann 2015 [58], Vanmechelen 2017 [51], Popadic 2011 [50], Morse 2007 [60], Yamauchi 2017 [28] | - | - |  |
| **segmentation on a single slice** | Marcon 2015 [9], Tracy 2003 [26], Lehtinen 2003 [56] | Lehtinen 2003 [56] | Lehtinen 2003 [56] |  |
| **DPSO method** | Sudhoff 2009 [64], Nordez 2009 [27], Amabile 2016 [53], Moal 2014 [59], Jolivet 2014 [68] | Sudhoff 2009 [64], Moal 2014 [59] | Moal 2014 [59] |  |
| **Automatic segmentation methods** | Engstrom 2011 [67], Andrews 2015 [65], Le Troter 2016 [48], Kim 2017 [29] |  |  |  |
